# EEG-Based Hypo-vigilance Detection Using Convolutional Neural Network

**DOI:** 10.1007/978-3-030-51517-1_6

**Published:** 2020-05-31

**Authors:** Amal Boudaya, Bassem Bouaziz, Siwar Chaabene, Lotfi Chaari, Achraf Ammar, Anita Hökelmann

**Affiliations:** 8 Digital Research Centre of Sfax, Sfax, Tunisia; 9 Institut Mines-Télécom, CNRS, Paris, France; 10 Université de Sherbrooke, Sherbrooke, QC Canada; 11 Digital Research Centre of Sfax, Sfax, Tunisia; 12 University of Sfax, Sfax, Tunisia; 13 Multimedia InfoRmation Systems and Advanced Computing Laboratory (MIRACL), University of Sfax, 3021 Sfax, Tunisia; 14 Digital Research Center of Sfax, B.P. 275, 3021 Sakiet Ezzit, Sfax, Tunisia; 15 University of Toulouse, IRIT-ENSEEIHT, Toulouse, France; 16 Institute of Sport Science, Otto-von-Guericke University Magdeburg, 39104 Magdeburg, Germany

**Keywords:** Hypo-vigilance detection, EEG, CNN

## Abstract

Hypo-vigilance detection is becoming an important active research areas in the biomedical signal processing field. For this purpose, electroencephalogram (EEG) is one of the most common modalities in drowsiness and awakeness detection. In this context, we propose a new EEG classification method for detecting fatigue state. Our method makes use of a and awakeness detection. In this context, we propose a new EEG classification method for detecting fatigue state. Our method makes use of a Convolutional Neural Network (CNN) architecture. We define an experimental protocol using the Emotiv EPOC+ headset. After that, we evaluate our proposed method on a recorded and annotated dataset. The reported results demonstrate high detection accuracy (93%) and indicate that the proposed method is an efficient alternative for hypo-vigilance detection as compared with other methods.

## Introduction

Hypo-vigilance has been one of the major causes of accidents in many areas such as driving [1], aviation [2] and military sector [3]. Hence, the drowsiness problem has gained great interest from researchers. This is today a real up to date problem within the current Covid-19 [4] pandemic where medical stuff is generally overbooked. In fact, the drowsy condition is expressed predominantly by the emergence of various behavioral signs such as heaviness in terms of reaction, reflex reduction, occurrences of yawning, heaviness of the eyelids and/or the difficulty of keeping the head in the frontal position relative to the field of vision. Many studies [5–8] have been proposed to detect hypo-vigilance based on biomedical signals such as electroencephalogram (EEG), electrocardiogram (ECG), electromyogram (EMG), and electrooculogram (EOG). Given, its high temporal resolution, portability and reasonable cost, the present work focus on hypo-vigilance detection by analyzing EEG signal of various brain’s functionalities using fourteen electrodes placed on the participant’s scalp. On the other hand, deep learning networks offer great potential for biomedical signals analysis through the simplification of raw input signals (i.e., through various steps including feature extraction, denoising and feature selection) and the improvement of the classification results.

In this paper, we focus on the EEG signal study recorded by fourteen electrodes for hypo-vigilance detection by analyzing the various functionalities of the brain from the electrodes placed on the participant’s scalp.

Various deep learning architectures [9] exist such as Convolutional Neural Network (CNN), Recurrent CNN (R-CNN), Auto-Encoder (AE), Deep Belief Network (DBN), including Long Short-Term Memory (LSTM) and Gated Recurrent Units (GRU). As in [10], the CNN architecture is the most used to biomedical signals analysis providing a high classification accuracy. Previous related work [11] proposes a hypo-vigilance detection method using CNN by facial features. This method showed a classification accuracy of 92.33%. Likewise [12], introduces an adaptive conditional representation learning system for driver drowsiness detection based on a 3D-CNN. The proposed system consists of four steps (spatio-temporal representation, data preprocessing, features combination and somnolence detection). The experimental results show a detection accuracy equal to 92.04%. In this paper, we propose a CNN hypo-vigilance detection method using EEG data in order to classify drowsiness and awakeness states. Accordingly, the proposed approach including used equipment are presented in Sect. 2. Section 3 describes the experimental results and the evaluation of the employed method. Finally, a conclusion and future work are drawn in Sect. 4.

## Proposed Approach

**Fig. 1.**
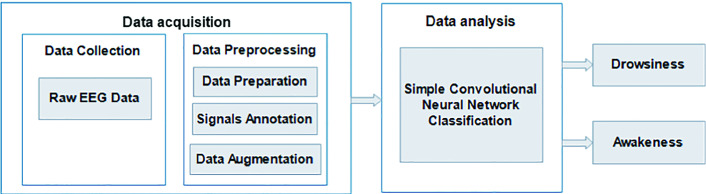
Pipeline for the proposed approach.

As shown in Fig. 1, the realization of the proposed approach is suggested by two primary procedures: data acquisition and data analysis. The following subsections provide a detailed explanation of each procedure.

### Data Acquisition

The EEG data acquisition procedure is made up of two main steps which are data collection and data preprocessing.

**Fig. 2.**
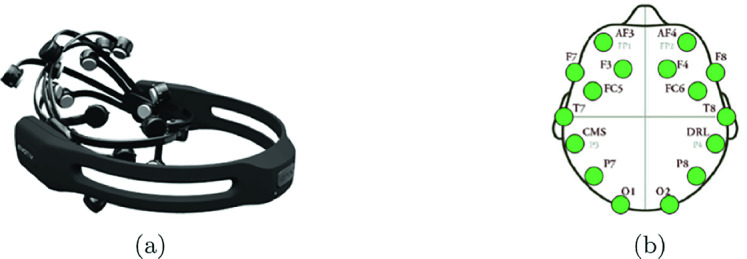
(a) Emotiv EPOC+ helmet, (b) Location of the Emotiv EPOC+ helmet electrodes (10–20 International Standard).

***Data Collection:*** To collect the raw EEG data from participants, we use an Emotiv EPOC+ headset as shown in Fig. 2[a] for the data acquisition process. The key feature of this headset is a non-invasive Brain computer Interface (BCI) tool designed for the development of human brain and contextual research [13].

The Emotiv EPOC + helmet contains fourteen active electrodes with two reference electrodes (DRL and CMS), as shown in Fig. 2[b]. The electrodes are placed around the participant’s head in the structures of the following zones: frontal and anterior parietal (AF3, AF4, F3, F4, F7, F8, FC5, FC6), temporal (T7, T8) and occipital-parietal (O1, O2, P7, P8).

***Data Preprocessing:*** The specific preprocessing steps of the data revolve around the following points which are data preparation, data annotation and data augmentation.

- **Data Preparation**
  During data acquisition, our raw EEG signals may be influenced by various sources of artifacts and noise such as endogenous electrical properties, specific fabrics physical structure, dipolar size variation, muscle shifts and Blinks. Hence, data processing is a preliminary step to denoising the raw signals. We suggest using an infinite impulse response (IIR) filter that manages an impulsive signal within time and frequency domains. Other sophisticated denoising approaches could be considered at the expense of higher computational complexity [14, 15].
- **Signals Annotation**
  To evaluate each individual’s state of exhaustion, we concentrate on the brain areas that are responsible for hypo-vigilance detection. In this regard, different brain waves are targeted such as [16]:
  - **Delta waves** refer to consciousness, sleep or deep sleep states. These waves were found in the temporal and occipital conditions with low frequency (less than 4 Hz) and high amplitude.
  - **Theta waves** design the relaxation and hypnosis states with a range of frequency between 4 and 8 Hz. Theta waves are extracted from the temporal zone and are produced during the first phase of slow sleep or in deep relaxation state.
  - **Alpha waves** refer to waking but relaxed states. These waves are captured in the posterior part, precisely the occipital region, with a frequency interval between 8 and 12 Hz and a low amplitude interval between 20 and 60 $\upmu$V.
  - **Beta waves** relate to alertness states. These waves are captured from the temporal and occipital lobes of the brain. They are characterized by high frequency interval of 12 to 30 Hz with a low amplitude interval of 10 to 30 $\upmu$V.
  - **Gamma waves** refer to hypervigilance states with a frequency interval between 30 to 80 Hz.

In the data annotation step, we only use the O1 and O2 electrodes of occipital zone which are responsible for drowsiness sensation.

**Fig. 3.**
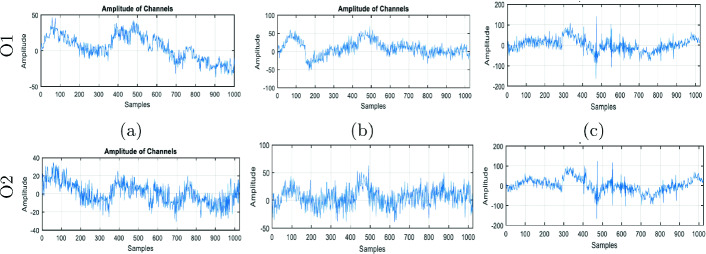
The monitoring of O1 and O2 electrodes in the mornings (a), afternoons (b) and evenings (c).

As an annotation example, Fig. 3 indicates the amplitudes of the alpha and theta signals from the two O1 and O2 electrodes reported for a participant in three periods of the day. The relaxation state has been indicated by alpha waves which have a frequency interval between 8 to 12 Hz and an amplitude interval between 20 to 60 $\upmu$V. The somnolence state has been indicated by theta waves which have a frequency interval between 4 to 8 Hz and an amplitude interval between 50 and 75 $\upmu$V.

- **Data augmentation**
  In order to reduce overfitting and increase testing accuracy, we use the data augmentation technique [17] which consists of increasing the training set by label-retaining data transformations. The purpose procedure is to extend the data by doubling the vectors from (5850, 2) to (59053, 2) where 5850 (resp. 59053) represents the vector size and 2 represents the class number.

### Data Analysis: Simple CNN Classification

**Fig. 4.**
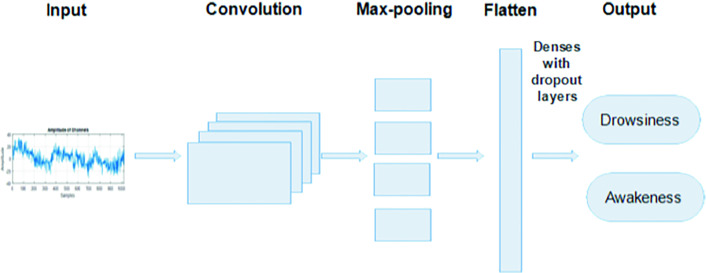
The diagram of the simple CNN used in the proposed approach.

The diagram of the neural network simple CNN used in our EEG drowsiness detection approach is represented in Fig. 4. The proposed simple CNN model is composed of the following six main layers:

- **The convolutional layers** allow the filter application and the features extraction characteristics of the input signals.
- **The sample-based discretization max-pooling-1D blocks** is used to sub-sample each input layer by reducing its dimensionality using a decrease in the number of the parameters to learn, there by reducing calculation costs.
- **The flatten layer** is used to flatten out multidimensional data.
- **The dropout layers** help to reduce the loss accuracy by regularizing and enhancing the overfitting of neural networks during the classification process.
- **The BatchNormalization layers** are used to scale and speed up learning of all activations. These layers normalize the previous activation layer output by subtracting the batches average and dividing it by the standard deviation to improve a neural network’s stability.
- **The dense layers** allow to done a connectivity function between the next and intermediate neurons layer.

## Experimental Evaluation

Our protocol revolves around the following axes: eight volunteers in which four women and four men aged twenty six and fifty eight with normal mental health. For each participant, we make three recordings of sixteen minutes divided over three day periods (morning, afternoon and evening). To fully understand the condition of the participants, we split the signal into windows to accurately identify these different states.

In the proposed simple CNN architecture for EEG signals classification, we use the Keras deep learning library. The different parameters as filters, kernel-size, padding, kernel-initializer, and activation of the four convolutional layers have the same values respectively 512, 32, same, normal and relu. The parameter values of the remaining layers are detailed in the following:

- the dropout layer value equal to 0.2 (respect. 0.5) is used to inactivate 20% (respect. 50%) of neurons in order to prevent overfitting.
- the Max-Pooling 1D layer is used with a filter size of 128.
- The muti-dimensional data output flatting using 1D flatten layer.
- For better classification results, two dropout layers are used. The first hidden layer takes a value of 128 neurons. Since a binary classification problem, the second layer takes a value of 1.

The choice of the optimization algorithm makes the difference between good results in minutes, hours or even days. There are various optimizers like Adam [18], SGD [19] and RMS pop optimizer [20]. In our model, we use the SGD optimizer which is more popular [21]. The method of this optimizer is simple and effective for finding optimal values in a neural network. Table 1 presents the hyperparameters choice of our model.

**Table 1.** Hyperparameters choices.

Parameters	Value
Optimization algorithm	SGD
Momentum	0.5
Batch size	64
Activation function	Sigmoid

For selecting the best accuracy rate of the proposed method, we propose to compare different results recorded by different numbers of electrodes. In [22, 23], the authors discover that the prefrontal and occipital cortex are the most important channels to better diagnose the hypo-vigilance state. In this regard, we choose the following recorded data:

- Recorded data by 2 electrodes (O1 and O2) electrodes from the occipital area.
- Recorded data by 4 electrodes (T7, T8, O1 and O2) from temporal and occipital areas.
- Recorded data by 7 electrodes (AF3, F7, F3, T7, O2, P8, F8) from prefrontal and occipital areas.
- Recorded data by 14 electrodes.

**Fig. 5.**
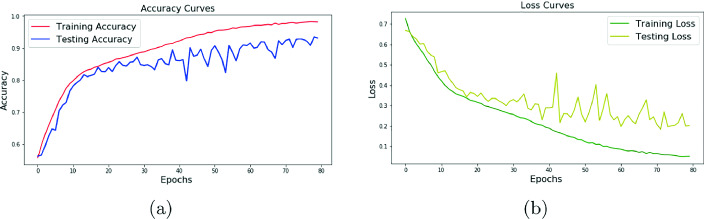
(a) Accuracy graph, (b) Loss graph.

For the distribution of our data, we choose 70% for the train part and 30% for the test. Table 2 presents the reported testing and training accuracy respectively with two, four, seven and fourteen electrodes. After convergence the optimum number of test epochs for all the different electrodes results establish a value equal to 80. The best results are given by the recording of 2 electrodes from the occipital area. The curves of testing and training results for recorded data by O1 and O2 electrodes are represented in Fig. 5.

**Table 2.** Training and testing results of the different numbers of electrodes with data augmentation.

Number of electrodes	2	4	7	14
Accuracy train	98.18%	98.28%	98.99%	98.99 %
Accuracy test	93.94%	65.58%	76.43%	77.43 %

According to results obtained in Fig. 5, we note that the test accuracy increases after a certain number of epochs and the test loss decreases. To test our system’s efficiency we measured the precision, recall and F1-score. Table 3 shows these different measures in our experimental configuration.

**Table 3.** Accuracy, precision, recall and F1-score of our experimental configuration

Accuracy	Precision	Recall	F1 score
93.94%	87.29%	99.79%	93.12%

For comparison purposes, we compare the proposed method with recent drowsiness methodology [24] where the authors propose a driver hypovigilance detection using the Emotiv EPOC+ helmet. The Common Spatial Pattern (CSP) algorithm is used for optimization accuracy of Extreme Learning Machine (ELM). The reported values in Table 4 indicate that our method gives the optimum accuracy value classification.

**Table 4.** Accuracy comparison with related works.

Drowsiness detection methodology	Accuracy	Classification method
R. Osmalina et al. [24]	91.67%	CSP algorithm
Proposed method	**93.94 %**	CNNs

## Conclusion

The present work proposes a CNN based approach for Hypo-vigilance detection. In order to create a EEG dataset, we recorded raw EEG data using Epoc+ headset. The suggested system achieves an average classification accuracy to 93.94% by testing it on a real dataset of eight participants. In future work, we will focus to improve classification accuracy with large datasets. Additionally, fusion with other biomedical signals should be also considered to improve the classification accuracy.
